# Risk of Symptomatic Infection after Non-Primary Congenital Cytomegalovirus Infection

**DOI:** 10.3390/microorganisms8050786

**Published:** 2020-05-25

**Authors:** Alessandra Coscia, Agata Leone, Carlotta Rubino, Ganna Galitska, Matteo Biolatti, Enrico Bertino, Chiara Peila, Francesco Cresi

**Affiliations:** 1 Neonatal Care Unit, Department of Public Health and Pediatric Sciences, University of Turin, 10126 Turin, Italy; alessandra.coscia@unito.it (A.C.); agata.leone@unito.it (A.L.); enrico.bertino@unito.it (E.B.); chiara.peila@unito.it (C.P.); francesco.cresi@unito.it (F.C.); 2 Laboratory of Pathogenesis of Viral Infections, Department of Public Health and Pediatric Sciences, University of Turin, 10126 Turin, Italy; ganna.galitska@unito.it (G.G.); matteo.biolatti@unito.it (M.B.)

**Keywords:** CMV, cCMV, SNHL, primary and non-primary CMV infection, maternal immunity

## Abstract

Cytomegalovirus (CMV) is the leading cause of congenital infection. Its occurrence is phenotypically heterogeneous. The type of maternal infection, primary or non-primary, is an important factor related to the symptomatic disease, the primary infection was long considered the only cause of severe neonatal disease. We aimed to analyze the association of primary and non-primary infection with pathological outcomes in infants and with long-term sequelae at follow-up. This was a monocentric retrospective observational study on a population of 91 infants diagnosed with a CMV infection at the Neonatal Care Unit of Neonatology at the Sant’Anna Hospital of Turin during the period of June 2005 to December 2018. Infants underwent clinical, laboratory, and neuroradiological evaluations at birth. Subsequently, the patients were monitored in an auxological, neurodevelopment, and audiological follow-up. Regarding primary vs. non-primary infection, we found a higher percentage of incidence of symptomatic and neurological localized infection, as well as long-term sequelae in the latter. However, no significant difference between the two populations was found. We underline the possibility of re-infection in previously immunized mothers (non-primary infection) with unfavorable neonatal and long-term outcomes.

## 1. Introduction

Cytomegalovirus (CMV) is the leading cause of congenital infection, affecting 0.5%–5% of live births worldwide [1]. Since CMV can cause severe auditory and neurobehavioral sequelae, it has an important impact on global health. In Italy, the prevalence of congenital cytomegalovirus (cCMV) infection is among the lowest in the literature and it varies between 0.15% in children born to women over 24 years of age and 0.51% of women below this age [2]. cCMV is a pathology with large phenotypic variability that manifests with a wide variety of signs and symptoms; the infected newborn can be either completely asymptomatic or show alterations of laboratory tests or display numerous symptoms from pathologies of the gastrointestinal tract and spleen up to neurological and cognitive disabilities. cCMV represents one of the main causes of sensorineural hearing loss (SNHL) [3].

The underlying causes of this variability are not yet fully known. One of the main variables is the trimester of infection as the main phase at risk for the development of neurological sequelae in infected infants [4]. When accurately determined, the type of infection enables defining the risk of its transmission to the fetus and, importantly, estimating the severity of the disease that can be assessed both as a symptom upon birth and as a long-term outcome. For a long time, primary maternal infections were assumed to have a more significant global clinical impact than non-primary infections [5,6]; however, more recent studies confirmed that congenital infections are also detected in newborns born from mothers with previous immunity. Maternal immunity does not seem to be protective neither in terms of preventing the transmission of the virus to the fetus nor in terms of the severity of the infection itself [7,8,9].

For these reasons, universal CMV screening of pregnant women is not recommended according to the latest consensus recommendations [3]. In the absence of a valid vaccine and with many vaccine candidates currently under development, the only means of prevention are hygiene measures [10]. Given the growing evidence of the relevance of non-primary infection, these measures should be directed to all pregnant women, both seronegative and seropositive [11]. 

The aim of our study was to analyze the association of primary and non-primary infection with pathological outcomes in infants with auxological, neurodevelopmental, and audiological sequelae at the follow-up to ensure appropriate evaluation of CMV risk in these subjects. 

## 2. Materials and Methods 

### 2.1. Study Design and Population Selection

This monocentric retrospective observational study was comprised of a population of 91 newborns diagnosed with congenital CMV infection enrolled at the Neonatal Care Unit of Sant’Anna University Hospital of Turin from June 2005 to December 2018. Ethics approval was provided on 8 April 2019 by the Ethical Committee of the City of Health and Science Hospital of Turin (Italy), protocol number 0041678. Informed consent was obtained from the parents of the children recruited into this study.

### 2.2. Study Population

Congenital CMV (cCMV) infection was confirmed based either on detection of CMV DNA in urine samples collected within the 21st day of life or on viral DNA detection on a Guthrie Card after the 21st day of life [12,13]. Symptomatic cCMV infection was defined if one or more of the following signs or symptoms were present: hematologic (thrombocytopenia, petechiae, splenomegaly), hepatic (hepatomegaly, hepatitis—defined as elevated transaminases or prolonged jaundice and/or elevated conjugated bilirubinaemia), auxological (small for gestational age (SGA)–defined as a birthweight <10th percentile compared to gestational age curves, microcephaly—defined as head circumference <3rd percentile compared to gestational age curves [14]), neurological (chorioretinitis, sensorineural hearing loss (SNHL), lethargy, convulsive crisis), ultrasound (US) abnormalities (ventriculomegaly, cysts, vasculitic striae, brain calcifications), and magnetic resonance imaging (MRI) abnormalities (cysts, myelination delay, ventriculomegaly, changes in the corpus callosum, hemispherical and/or cerebellar asymmetries, alterations of auditory courting). 

In each case of this study, the data on the maternal infection were analyzed. Primary infection was defined as a seroconversion in pregnancy in previously seronegative women. We were able to date the trimester of infection by identifying the date of maternal seroconversion. Otherwise, non-primary infection was defined as a re-infection in previously seropositive women. In this case, tracing the history of maternal suggestive symptoms during pregnancy and fetal abnormalities enabled us to identify the trimester of infection.

### 2.3. Follow-Up

After discharge, patients underwent follow-up visits to evaluate the evolution of the pathologies. Auxological, neurodevelopmental, and audiological follow-up was performed. Follow-up lasted 12 months for asymptomatic infants with an asymptomatic disease course and 24 months for both symptomatic infants and those who manifested abnormalities during follow-up. 

As part of the auxological follow-up, the weight, length, and head circumference parameters were measured during each visit. The measurements were then plotted to the corresponding Centers for Disease Control and Prevention (CDC) growth charts and the centiles were calculated [15]. The weight values under the 3° centile on CDC curves divided for age and sex at the last visit performed were defined as a display of auxological impairment. 

Neurodevelopmental follow-up was performed using the *Griffiths Mental Development Scale II*. At the end of the evaluation, the General Quotient was obtained. In our study, a General Quotient <85 was considered as neurodevelopmental delay. 

Evaluations of the audiological follow-up were performed at the first, second, third, and sixth audiological visits. The subject was diagnosed as hypoacusic when presented with auditory brainstem response (ABR) values >30 or auditory steady state response (ASSR) values >30, separately for each ear. Indication of the need for cochlear implant, used as a proxy of bilateral severe SNHL, at the end of the audiological follow-up period was also determined as a long-term outcome. 

### 2.4. Statistical Analysis 

A statistical analysis was performed using R (www.r-project.org). Results for continuous variables are provided as means and standard deviation. Results for factorial variables are given as numbers and percentages. The categorical variables between groups were compared using a series of Fisher’s exact tests. Values of *p* < 0.5 were considered statistically significant.

## 3. Results

Of the 91 children diagnosed with congenital CMV infection, 41 women (45%) and 50 men (55%) were enrolled in the present study. The baseline characteristics of our selected population are summarized in Table 1.

Regarding the trimester of cCMV infection during pregnancy, the following values were obtained: the data on 39 patients were missing, whereas the determined cases accounted for 40% of patients infected in the first trimester, 29% in the second trimester, and 16% in the third, accordingly. The data regarding the type of infection were distributed as follows: the data on 42 patients were missing, the determined cases included 86% of patients with primary infection and 14% with non-primary infection. The characteristics of maternal infection are summarized in Table 2. 

Characteristics of fetal abnormalities and symptoms of infection detected at birth are summarized in Table 3. 

Considering data of neurodevelopmental, auxological and audiological follow-up, simplified in binomial variables, the percentages concerning the long-term sequelae driven by cCMV were calculated. While the auxological delay has been reported in 25% of children, 31% displayed neurodevelopmental impairment, 21% displayed hearing loss, and 9% of patients indicated a need for cochlear implantation used as a proxy of severe bilateral SNHL. 

When analyzing the different occurrences in the first trimester vs. the second and third trimesters of infection, we found that in the first trimester, the occurrence of a symptomatic infection at birth accounted for 81% vs. 66% in the other trimesters and with no significant difference in the two groups (*p* = 0.346). However, the occurrence of a neurologically symptomatic infection was 75% vs. 40% (*p* = 0.021). Regarding long-term follow-up, we found that in the first trimester, the occurrence of need for a cochlear implant was 28% vs. 0% (*p* = 0.003). Other data on the differences between the two patients’ groups are summarized in Table 4. 

Particular attention was paid to the non-primary infection and its incidence in symptomatic cases in this patients’ group is reported in Table 5. No significant differences between the two groups were found. 

Finally, in Table 6, we report the series of our cases of infants affected by cCMV after maternal non-primary infection. 

## 4. Discussion

Cytomegalovirus is the leading cause of congenital infection worldwide and it represents an important global health burden. Considering the extreme variability of disease manifestations, identifying congenital CMV infection at risk implies a significant step toward the improvement of the patients’ management, treatment, and follow-up.

In this retrospective observational study, we analyzed auxological, neurodevelopmental, and audiological follow-up outcomes of infants diagnosed with congenital CMV to improve the understanding of the clinical course of the infection, as well as to identify the factors defining what population may appear most at risk of presenting the CMV disease. 

The data regarding the trimester of infection demonstrated that the infants whose mothers were infected during the first trimester presented neurological signs more often (75% vs. 40% (*p* = 0.021)). These results are in line with those reported in the literature [1], highlighting the actual need to diagnose and to keep the first trimester infections under strict control. We point out the significant difference in the incidence of the indication of a need for a cochlear implant in the two population groups: 28% in the first trimester and 0% in the others (*p* = 0.019), suggesting that severe bilateral SNHL is limited only in the former. In our study, we focused on outcomes within the first/second year of life, the most critical periods for the child neurodevelopment. Aware of the possibility of late-onset SNHL [16,17], it would be interesting to analyze, in further works, how many of these children have a need for belated hearing aid repair.

Considering the differences in the type of infection analyzed (primary vs. non-primary), the percentage of symptomatic cases at birth was 71% for primary and 85% for non-primary patients, whereas neurological localization was reported in 54% of primary and 85% of non-primary cases. The same tendency was observed for long-term sequelae, in which the non-primary infection appeared to have an increased risk of unfavorable auxological, neurodevelopmental, and audiological outcomes. No significant differences were found between the two patients’ groups. However, these last results may be explained by the group of patients with primary infection being composed of 42 individuals, whereas the non-primary group included seven individuals, implying a low statistical significance. Notably, the data on the type of CMV infection were missing 42 patients out of 91. This was due to the absence of universal screening in pregnancy. Thus, it is often impossible to trace the exact characteristics of maternal infection. 

Although our study design did not allow us to provide essential epidemiological information, our results showed that non-primary CMV infection must not be underestimated even though the percentages found are unlikely to be generalized to the entire population. In this type of infection, we mostly likely only diagnosed and selected infants with pathologies and, therefore, missed the diagnosis of the asymptomatic ones. For patients with a primary infection, we obtained a significant heterogeneous cohort.

The presence of cases with important clinical sequelae in a subgroup of children born to mothers with previous immunity to CMV, for which we underline the incidence of the need for a cochlear implant, contradicts the reported cases in the literature. Thus, highlighting this type of infection as of particular importance in diagnostics and clinical management supporting the opinion of several authors that non-primary CMV infection is at least as serious as a primary infection [7,8,9]. Consequently, prevention measures should also be extended to seropositive mothers and particular attention must be paid to all early signs of prenatal and neonatal CMV infection in these infants. 

## Figures and Tables

**Table 1:** Baseline characteristics.

**Birth**	***N* = 48**
Eutocic	26 (53%)
Cesarean	23 (47%)
**Gestational Age**	***N* = 90**
	37.9 (±1.94)
Apgar 5′ ≥ 7 *	***N* = 50**
	50 (100%)
**Birth weight**	***N* = 89**
	2786 ± 658 g
**Birth head circumference**	***N* = 89**
	33 ± 2.2 cm

* Apgar score: Evaluation of the heart rate, muscle tone, grimace, color, and breathing in the first minutes of life. A score of 7 to 10 after 5′ is considered normal.

**Table 2:** Characteristic of maternal infection.

**Infection Diagnosis**	***N* = 71**
Not detected in pregnancy	17 (24%)
Targeted screening	35 (49%)
Intrauterine growth restriction (IUGR)	4 (6%)
Fetal ultrasonography abnormalities (non-IUGR)	3 (4%)
Flu-like syndrome	12 (17%)
**Trimester of Infection**	***N* = 52**
I	21 (40%)
II	15 (29%)
III	16 (31%)
**Type of Infection**	***N* = 49**
Primary	42 (86%)
Non-primary	7 (14%)

**Table 3:** Fetal and neonatal infection features.

**Fetal Ultrasonography Abnormalities**	***N* = 91**
Not specified	6 (7%)
Ventriculomegaly	4 (4%)
Frontal cyst	1 (1%)
IUGR	12 (23%)
**Signs at Birth**	***N* = 91**
Small for Gestational Age (SGA)	35 (40%)
Thrombocytopenia	5 (9%)
Neutropenia	11 (19%)
Impaired liver function	9 (17%)
Aspartate Aminotransferase/Alanine Aminotransferase (AST/ALT) Alteration	10 (19%)
Gamma-Glutamyl Transpeptidase (GGT) Alteration	20 (37%)
Petechiae	1 (1%)
Hepatomegaly	3 (4%)
Splenomegaly	2 (3%)
Chorioretinitis	1 (1%)
Seizures	0
Lethargy	0
Altered audiological screening	14 (17%)
Altered Brain Ultrasound	48 (56%)
Altered Brain Magnetic Resonance	19 (37%)

**Table 4:** Differences between trimester.

Outcomes	I *n* (%)	II/III *n* (%)	*p*-Value
Symptomatic infection	17 (81)	20 (66)	0.346
Neurological localized infection	15 (75)	12 (40)	0.021
Auxological Impairment	5 (28)	4 (18)	0.705
Neurodevelopmental impairment	4 (23)	2 (9)	0.373
Hearing Loss	7 (33)	3 (12)	0.149
Need for cochlear implant	6 (28)	0	0.003

**Table 5:** Differences between the two types of infection.

Outcomes	Primary *n* (%)	Non-Primary *n* (%)	*p*-Value
Symptomatic infection	30 (71)	6 (85)	0.658
Neurological localized infection	22 (54)	6 (85)	0.214
Auxological Impairment	9 (28)	2 (29)	1
Neurodevelopmental impairment	5 (15)	1 (20)	1
Hearing Loss	9 (24)	3 (50)	0.325
Need for cochlear implant	4 (10)	3 (43)	0.05

**Table 6:** Non primary infection.

Case	Trimester of infection	Outcomes
1	I	Severe bilateral Sensorineural Hearing Loss (SNHL); cyst on brain ultrasound (US) and MRI; Neurodevelopmental impairment
2	NA	SGA, cyst on brain US
3	I	Fetal US abnormalities, thrombocytopenia, widespread hyperechogenicity and germinal matrix hemorrhage on brain US
4	I	Asymptomatic
5	I	Fetal US abnormalities (ventriculomegaly), widespread hyperechogenicity and ventriculomegaly on brain US; cyst, delayed myelination, and ventriculomegaly on MR; macular abnormalities on fundus oculi and fluctuating severe SNHL
6	NA	IUGR, SGA, microcephaly, widespread hyperechogenicity on brain US
7	NA	IUGR, SGA, microcephaly, severe bilateral SNHL, cyst on brain US
